# Baicalein as Promising Anticancer Agent: A Comprehensive Analysis on Molecular Mechanisms and Therapeutic Perspectives

**DOI:** 10.3390/cancers15072128

**Published:** 2023-04-03

**Authors:** A K M Helal Morshed, Supti Paul, Arafat Hossain, Tuli Basak, Md. Sanower Hossain, Md. Mehedi Hasan, Md. Al Hasibuzzaman, Tanjim Ishraq Rahaman, Md. Abdur Rashid Mia, Pollob Shing, Md Sohel, Shabana Bibi, Dipta Dey, Partha Biswas, Md. Nazmul Hasan, Long Chiau Ming, Ching Siang Tan

**Affiliations:** 1 Pathology and Pathophysiology, Academy of Medical Science, Zhengzhou University, No. 100 Science Avenue, Zhengzhou 450001, China; 2 Department of Chemistry, University of Dhaka, Dhaka 1000, Bangladesh; 3 Biochemistry and Molecular Biology Department, Life Science Faculty, Bangabandhu Sheikh Mujibur Rahman Science and Technology University, Gopalganj 8100, Bangladesh; 4 Department of Genetic Engineering and Biotechnology, Faculty of Science and Engineering, East West University, Dhaka 1212, Bangladesh; 5 Centre for Sustainability of Ecosystem and Earth Resources (Pusat ALAM), Universiti Malaysia Pahang, Gambang, Kuantan 26300, Malaysia; 6 Institute of Nutrition and Food Science, University of Dhaka, Dhaka 1000, Bangladesh; 7 Department of Biotechnology and Genetic Engineering, Faculty of Life Science, Bangabandhu Sheikh Mujibur Rahman Science and Technology University, Gopalganj 8100, Bangladesh; 8 Department of Pharmaceutical Technology, Faculty of Pharmacy, International Islamic University Malaysia, Kuantan 25200, Malaysia; 9 Department of Genetic Engineering and Biotechnology, Faculty of Biological Science and Technology, Jashore University of Science and Technology, Jashore 7408, Bangladesh; 10 Department of Biochemistry and Molecular Biology, Primeasia University, Banani, Dhaka 1213, Bangladesh; 11 Department of Bioscience, Shifa Tameer-e-Millat University, Islamabad 44000, Pakistan; 12 Yunnan Herbal Laboratory, College of Ecology and Environmental Sciences, Yunnan University, Kunming 650091, China; 13 Laboratory of Pharmaceutical Biotechnology and Bioinformatics, Department of Genetic Engineering and Biotechnology, Jashore University of Science and Technology, Jashore 7408, Bangladesh; 14 School of Medical and Life Sciences, Sunway University, Sunway City 47500, Malaysia; longchiauming@gmail.com; 15 School of Pharmacy, KPJ Healthcare University College, Nilai 71800, Malaysia

**Keywords:** Baicalein, ROS, apoptosis, autophagy, flavonoids, anti-inflammatory, angiogenesis, nanomedicine, synergistic effects

## Abstract

**Simple Summary:**

Cancer is a multifactorial disease characterized by excessive cell proliferation in a specific organ. The most common treatments include chemotherapy, radiation therapy, surgery, hormone therapy, bone marrow transplantation, and immunotherapy. The aim of our study on Baicalein was to determine whether it has the potential for therapeutic effects, such as arresting cancer cell growth via the MAPK pathway and apoptosis through ROS, 12-lipoxygenase, and PI3K/Akt. We determined that Baicalein, a flavonoid extract isolated from dried roots, possesses anti-cancer properties, such as suppressing cell growth and differentiation, inhibiting metastasis, accelerating apoptosis, and elevating autophagy. Several in vitro and in vivo studies have demonstrated that it suppresses malignant cells by down-regulating anti-apoptotic proteins and up-regulating tumor suppressor p53. Further research should focus on improving the bioavailability of Baicalein using systems biology, RNA sequencing, proteomics, genomics, bioinformatics, and nano-medicine-based applications.

**Abstract:**

Despite significant therapeutic advancements for cancer, an atrocious global burden (for example, health and economic) and radio- and chemo-resistance limit their effectiveness and result in unfavorable health consequences. Natural compounds are generally considered safer than synthetic drugs, and their use in cancer treatment alone, or in combination with conventional therapies, is increasingly becoming accepted. Interesting outcomes from pre-clinical trials using Baicalein in combination with conventional medicines have been reported, and some of them have also undergone clinical trials in later stages. As a result, we investigated the prospects of Baicalein, a naturally occurring substance extracted from the stems of *Scutellaria baicalensis* Georgi and *Oroxylum indicum* Kurz, which targets a wide range of molecular changes that are involved in cancer development. In other words, this review is primarily driven by the findings from studies of Baicalein therapy in several cancer cell populations based on promising pre-clinical research. The modifications of numerous signal transduction mechanisms and transcriptional agents have been highlighted as the major players for Baicalein’s anti-malignant properties at the micro level. These include AKT serine/threonine protein kinase B (AKT) as well as PI3K/Akt/mTOR, matrix metalloproteinases-2 & 9 (MMP-2 & 9), Wnt/-catenin, Poly(ADP-ribose) polymerase (PARP), Mitogen-activated protein kinase (MAPK), NF-κB, Caspase-3/8/9, Smad4, Notch 1/Hes, Signal transducer and activator of transcription 3 (STAT3), Nuclear factor erythroid 2-related factor 2 (Nrf2)/Kelch-like ECH-associated protein-1 (Keap 1), Adenosine monophosphate-activated protein kinase (AMPK), Src/Id1, ROS signaling, miR 183/ezrin, and Sonic hedgehog (Shh) signaling cascades. The promise of Baicalein as an anti-inflammatory to anti-apoptotic/anti-angiogenic/anti-metastatic medicinal element for treating various malignancies and its capability to inhibit malignant stem cells, evidence of synergistic effects, and design of nanomedicine-based drugs are altogether well supported by the data presented in this review study.

## 1. Introduction

Cancer is a group of diseases defined by aberrant cell proliferation, which can invade or spread to different body parts [1]. Cancer is one of the leading causes of death worldwide and a major global health problem, and death and morbidity are escalating in both industrialized and developing regions. As a result, this public health burden needs to receive much greater attention. In wealthy countries, the survival rate of childhood cancer, in particular, has substantially increased over time, related to economic, environmental, and genetic factors. Still, it remains low in poor and middle-income countries [2]. Recent data suggest that tobacco use accounts for roughly 22% of cancer fatalities [3], and obesity, poor nutrition, lack of physical exercise, and excessive alcohol consumption account for 10% [4]. 

Extraordinary improvements in the detection and treatment of cancer have been made possible by developments in science and technology. An estimation showed that two out of every five people would develop cancer at some point [5]. The American Cancer Society evaluates cancer cases and related reports annually in the US, and its most recent estimation in 2021 publicized 1,898,160 cases with 608,570 mortalities [6]. According to the Global Cancer Observatory, owned by WHO, the highest cancer rate was found in Hungary at 371 cancer patients per 100,000 people. In Asia, the Republic of Korea (314 cases per 100,000 population) has the highest reported cancer incidence [7]. 

Most tumors remain an insurmountable challenge to eradicate for the modern medical system. Today, surgery, radiation, chemotherapy, and immunotherapy are the main therapeutic modalities for most malignancies. Due to the severe side effects of chemotherapy medications and the prevalence of numerous types of drug resistance, therapeutic effects are severely diminished despite the alternative therapies. The treatment type depends on the tumor’s location, grade, and sickness stage, as well as the patient’s general condition [8]. A variety of cancer therapies in the experimental phase are also being developed.

Recent research has identified traditional Chinese medicines as a new source of anti-cancer medications to lessen the side effects of cancer chemotherapies. Natural agents provide various benefits, including excellent price, accessibility, and lower toxicity. In today’s market, there are four types of plant-derived anti-cancer agents available: taxanes (paclitaxel and docetaxel), epipodophyllotoxins (etoposide and teniposide), camptothecin derivatives (camptothecin and irinotecan), and the vinca alkaloids (vinblastine, vincristine, and vindesine) [9]. Disease prevention and treatment have traditionally been accomplished by using herbal plants. Many individuals still use herbal nutraceuticals as their primary form of medicine. More than half of the medications used in clinical trials are made from natural products. Many researchers have looked into numerous herbal remedies and natural products in cancer treatment in recent years [10]. A lot of evidence shows the excellent efficacy of Baicalein in treating and preventing many types of cancer.

Baicalein, a flavonoid extract (5,6,7-trihydroxy-2-phenyl-4H-1-benzopyran-4-one) derived from the dried root of *Scutellaria baicalensis* Georgi, can inhibit cancer-promoting mechanisms such as metastasis, angiogenesis, and inflammation without harming healthy cells [11]. Despite having enormous prospects for anti-cancer use, low bioavailability limits its applications. Baicalin is a glycoside, a compound that results from the combination of Baicalein and glucose, and is more water soluble than Baicalein with greater bioavailability. However, the anticancer and anti-angiogenesis efficacy of Baicalin is lower than that of baicalein [12]. More specifically, the advancement is that the target mechanisms of signaling pathways of baicalein’s anti-cancer potential have been well developed. Baicalein’s anti-tumor properties mostly rely on its ability to inhibit numerous complex cascades. Baicalein acts on cyclins that control the cell cycle, oxidative radical scavenging, mitogen-activated protein kinase (MAPK), protein kinase B (Akt), mammalian target of rapamycin (mTOR), MMP-2/-9 (matrix metalloproteinase-2/-9) expression, and caspase-9/-3 activation. This induces apoptosis and inhibits tumor invasion, metastasis, and progression [13]. It has been utilized as an antioxidant, anti-viral, anti-bacterial, anti-inflammatory, anti-allergic, and other applications [14]. Additionally, Baicalein has been known to have anti-cancer properties for some time [15].

Baicalein exerts its actions via several biological processes, such as inhibition of cell proliferation, metastasis, angiogenesis, and inflammation and promotion of apoptotic cancer-cell death and autophagy [16]. Since cancer treatment methods include standard resection and chemotherapy, which carry a high risk of death, there is a lot of interest in locating a natural treatment that is reasonably non-toxic and may help to reduce side effects without compromising the therapeutic efficacy. Baicalein has the potential for such a role, according to numerous studies [17]. It inhibits cancer cell growth [18], promotes apoptosis [19], and causes cell cycle arrest in hepatocellular [15], human breast [20], myeloma [21], T24 bladder cancer cells [22], and prostate cancer [23]. Baicalein has shown considerable promise as a treatment for lung cancer in many studies [24].

Additionally, Baicalein prevented the growth of various chemotherapy cancers in mouse models [25]. When used in conjunction with chemotherapy, Baicalein could be established as a novel anti-cancer medication to treat cancers [26]. It has shown considerable promise in treating and preventing cancer with few side effects like constipation, stomach pain, and vomiting [27].

These findings suggest that Baicalein has the potential to be used in both the prevention and treatment of cancer and could be useful in the development of new drugs. This comprehensive review study addresses evidence-based investigations, particularly Baicalein’s ability to disrupt several molecular cascades, promising targets for cancer treatment. Additionally, this study demonstrates the numerous anticancer perspectives of Baicalein from published research studies. Many preclinical trials that provide evidence for its anticancer activity are also mentioned in this review.

## 2. Overview of Baicalein

**Figure 1:**
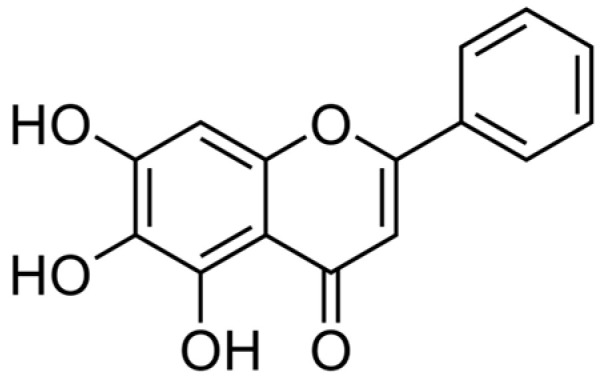
Chemical structure of Baicalein (Source: PubChem CID, 5281605).

As chemotherapeutic and nutritional chemopreventive agents, natural agents are gaining popularity. They have numerous benefits, including increased availability, cost-effectiveness, and reduced toxicity. Baicalein is an antioxidant sourced from the stem of *Scutellaria baicalensis* and *Oroxylum indicum* plants (generally known as Chinese Huang Qin) [10,28]. More than 50 flavonoids were isolated from the stems of *S. baicalensis* Georgi. Flavonoid consumption is linked to a lower risk of developing cancer, inflammatory processes, and cardiovascular disease. Baicalein has a crucial, intense characteristic feature responsible for its pharmacological activity [28,29,30,31]. Baicalein (C15H10O5), also known medically as 5,6,7- trihydroxyflavone (Figure 1), is a flavonoid substance with a polymer backbone of a two-phenyl-chromen-4-one (2-phenyl-1-benzopyran-4-one) [28]. The molecular weight of baicalein is 270.24 Da.

Relative to the colon, the gastric region and small intestine are better at absorbing baicalein. According to Biopharmaceutic Classification System (BCS) techniques, gut permeability steadily declined from the duodenal regions to the colonic part. Baicalein absorption in various gut regions could be subjected to passive transport processes and substantial metabolism, both in vivo and in vitro [32].

According to a laboratory study in a rat model, bile dramatically enhances the absorption of Baicalein [33]. Baicalein metabolites dominated the rats’ circulatory bloodstream following direct and parenteral bulk application [34]. After receiving oral medication, it was shown that intestinal flora was crucial in determining the distinct baicalein metabolites (Baicalin, Oroxylin A, Oroxin A, Chrysin, Baicalein-6-O-glucoside, Baicalein-6-O-glucuronide, Baicalein-6,7-di-O-glucuronide, Baicalein-sulfate, etc.) which were accessible in multiple digestive tract regions [34]. Those metabolites were found by some complex approaches that occurred in the gut mucosa, like glucuronidation, glycoxidation, methylation, and sulfation.

In addition to the gut, the liver substantially metabolizes Baicalein, which aids in pre-systemic metabolism [35]. A metabolic by-product known as 7-methoxybaicalein 6-O-glucuronide was found in a serum specimen following oral ingestion of baicalein granules in human subjects [36]. Significant glucuronides of baicalein, such as Baicalin, are produced in large quantities in the hepatocytes and intestinal microsomes of humans and mice.

Pre-systemic metabolism of Baicalein involves the enzyme UDP-glucuronosyltransferase. UDP-glucuronosyltransferase 1A9 was shown to have the maximum hepatic drug clearance and Vmax when calculated against other UDP-glucuronosyltransferases, making it the most effective in converting baicalein into Baicalin [37]. Baicalein is converted by catechol-O-methyltransferase to the methylated residue oroxylin A. Oroxylin A-7-O-β-D-glucuronide is produced by additional metabolic processing with UGT and is a component of human micturition [38].

Studies on Baicalein have indicated that it possesses multiple beneficial characteristics, such as being effective against oxidative stress, acute and chronic inflammation, malignancy, diabetes mellitus, and ulcerative colitis. Additionally, it has anti-thrombotic and anti-viral effects with cardioprotective, neuroprotective, eye-protective, and hepatoprotective features [39,40,41]. All of these functions are achieved by targeting a variety of critical signaling cascades. However, there is a paucity of information concerning these substances’ therapeutic applications and optimum dosages.

## 3. Molecular Pathway-Based Anticancer Properties of Baicalein

### 3.1. Baicalein Induces ROS for Cancer Treatment

Reactive oxygen species (ROS) are highly reactive oxygen-containing molecules that arise as natural by-products of normal metabolic processes, such as O_2_, HOCl, and OH, which can cause cellular damage if not appropriately regulated [42]. Once deemed hazardous by-products of mitochondrial function, ROS have been recently recognized for their important cellular roles in scavenging and signaling [43,44]. However, when ROS production becomes excessive, it can result in mitochondrial dysfunction, oxidative stress, and, ultimately, cellular apoptosis [45].

**Figure 2:**
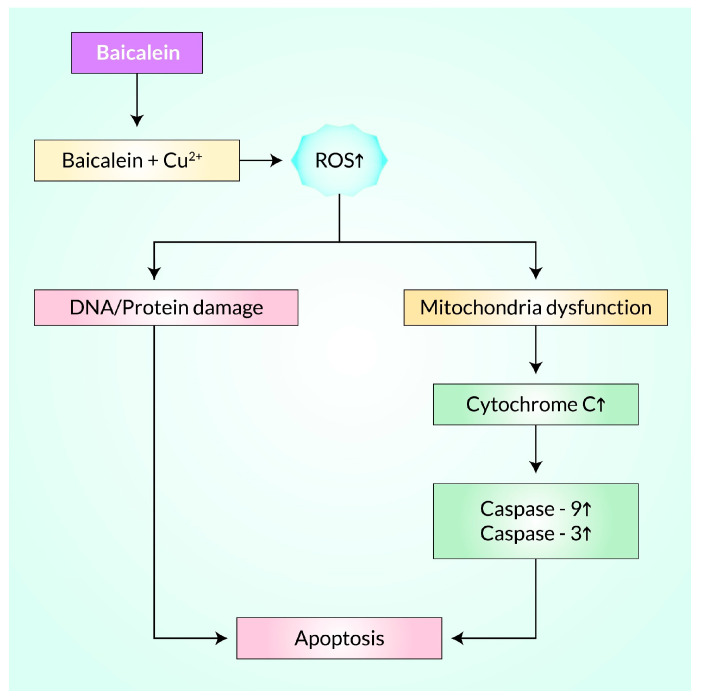
Predictive mechanism of Baicalein-induced apoptosis through regulating ROS signaling in cancer cell lines. This figure modified after Liu et al. [50].

A study demonstrated that Baicalein could induce growth inhibition in Hep G2 (hepatocellular carcinoma cell cluster) and MCF-7 (breast cancer cell cluster) cancer cells by regulating ROS via releasing H_2_O_2_ and capturing ·O_2_- radicals [46]. It significantly inhibited proliferation against breast cancer cells through multiple ROS-mediated pathways (Figure 2). Baicalein initially incited the formation of ROS, which subsequently aimed at endoplasmic reticulum stress and stimulated the Ca^2+^/-reliant mitochondrial death pathway. In this way, Baicalein reduced MMP levels, leading to cytochrome C release, increased caspase-3, and finally triggered cell death in MDA-MB-231 breast cancer cells [47] and SCC-4 human tongue cancer cells [48].

The effect of Baicalein on apoptosis in human bladder cancer 5637 cells was investigated, and it was found that it induces ROS generation. Pretreatment with the antioxidant N-acetyl-l-cysteine reduced caspase activation, suggesting that ROS plays a vital role in regulating caspase activation during Baicalein-induced apoptosis. That study demonstrated the positive relation of Baicalein-induced ROS generation with events of mitochondria that led to apoptosis. It can overcome tumor necrosis factor-related apoptosis-inducing ligand (TRAIL) resistance in cancer cells as significant cancer cells resist apoptosis induced by TRAIL. Through the production of ROS, Baicalein activates DR5 up-regulation and subsequently facilitates TRAIL resistance in PC3 cancer cells, which paves the way to overcome TRAIL resistance in mentioned cancer cells [49].

Baicalein inhibits the growth of human breast cancer MCF-7 cells by inducing mitochondrial apoptotic cell death. It does this by producing ROS, such as hydroxyl radicals, and reducing Cu (II) to Cu (I) in the Baicalein–Cu (II) system. These ROS lead to DNA and protein degradation [50]. In mitochondria-mediated apoptosis, Baicalein causes a significant increase in the expression of Bax and disruption in the expression of Bcl-2. This reduces mitochondrial membrane potential (ΔΨm) and the release of cytochrome C from mitochondria to the cytosol [51,52]. This, in turn, activates caspase -9 and caspase 3, ultimately resulting in apoptosis [53]. In summary, Baicalein induces ROS formation, which triggers mitochondrial apoptotic cell death and inhibits the growth of MCF-7 cells [50].

### 3.2. Baicalein Activates p53 in Cancer

The p53 gene is a type of tumor suppressor gene that produces the p53 protein when expressed. However, p53 is the most commonly mutated tumor suppressor gene of human malignancies, with over half of all human malignancies having inactivated p53 mutations. The p53 protein is found in the nucleus of cells and plays a critical role in regulating cell death and division. Alterations to the p53 gene can lead to uncontrolled cell proliferation and the spread of cancer throughout the body (https://www.ncbi.nlm.nih.gov/books/NBK22268/, accessed on 21 July 2022). To exert its anti-tumor effects, p53 inhibits different cyclins or cyclin-dependent kinases (CDKs) to modulate the cell cycle [54]. Other mechanisms include scavenging oxidative radicals, inhibiting MAPK, Akt, or mTOR activity, initiating apoptosis through upregulation of caspase-9/-3, and reducing the expression of MMP-2/-9 to repulse tumor invasion and metastasis [55,56,57].

**Figure 3:**
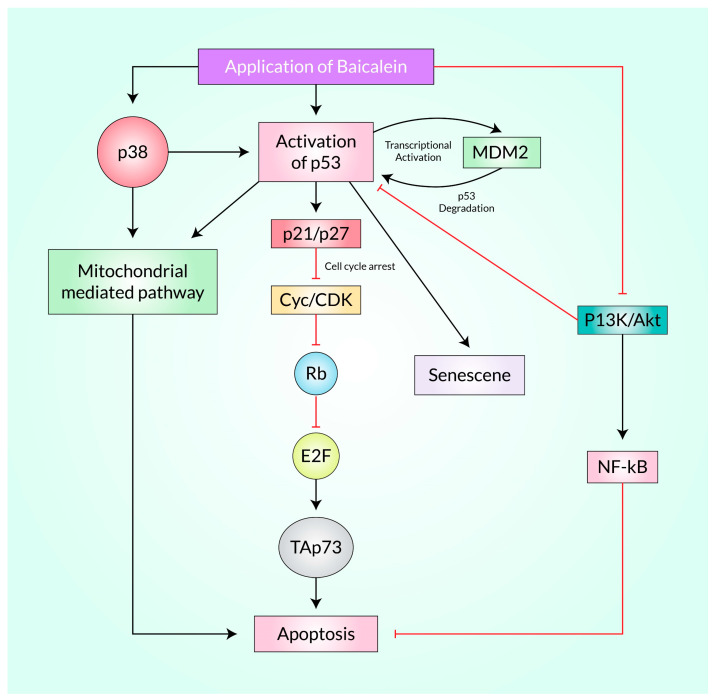
Baicalein increases the tumor suppressor proteins p38 and p53 and triggers apoptosis in tumor cells by inhibiting PI3K/Akt and subsequent proteins. Aditionally, this research highlights how Baicalein may impact cell cycle progression through the p53/Rb signaling pathway. Also, how MDM2-mediated degradation of p53 controls the senescence is depicted here. Here, TAp73 is a tumor suppressor and a structural homolog of p53. E2F is known as a transcriptional factor.

According to studies, p53 functions as a transcriptional activator of the CDK inhibitor p21, which negatively regulates the cyclin E-CDK2 complex and stops the cell from entering the S phase, resulting in cell cycle seizing at the G1 phase [58,59]. According to Ling et al. [60], Baicalein’s antiproliferative activity is mediated by the p53/Rb signaling axis (Figure 3). The amounts of proteins known to promote G1 shift, such as cyclin D and E, cdk2 and 4, and CDK blockers, such as p16, p21, and p27, were investigated in their experiment. The PKC (Protein kinase C) levels, p-Rb (Retinoblastoma Protein, a tumor suppressor protein), and p53, all recognized mediators of cell cycle arrest, were also investigated. Levels of p16, p21, p27, and p53 were up-regulated in a concentration-dependent way after Baicalein administration, whereas cyclin D and E, cdk2 and 4, and p-Rb levels were all down-regulated. According to the findings, the final suggestion is that Baicalein may influence cell cycle progression via the p53/Rb signaling pathway [60].

Qi et al. reported that Baicalein enhances miR-3178 expression, which reduces cancer progression, induces apoptosis, and disrupts cell cycles in HCC cells. Baicalein’s anti-cancer activity is rescued when miR-3178 is inhibited, and miR-3178 works against hepatocellular cancer by blocking HDAC10. Research has shown that Baicalein can influence the AKT/MDM2/p53 pathway by modulating the up-regulation of miR-3178 and Histone deacetylase 10 (HDAC10), which accelerates apoptotic cell death [61]. The MDM2-mediated breakdown is responsible mainly for keeping p53 at low cell concentrations. Therefore, interrupting the MDM2-p53 relationship to restore p53 function is an effective anti-cancer strategy. KEGG (Kyoto Encyclopedia of Genes and Genomes), a bioinformatic asset to study genes and genomes, reveals that MDM2 is one of the target genes of Baicalein (*p* < 0.05) [62].

Senescence is a cellular process that halts cell proliferation, in which senescent cells lose their ability to divide indefinitely. Senescent cells show morphological and metabolic alterations, as well as chromatin remodeling, modifications of gene manifestations, and the secretion of a pro-inflammatory phenotype known as the SASP (Senescence-associated secretory phenotype). MDM2-mediated degradation of p53 essentially controls senescence, which is crucial in preventing various disorders such as cardiovascular, nephrotic, and hepatic diseases, promoting organ rejuvenation and anti-cancer activity [63]. Treatment with Baicalein induces cell senescence in colon carcinoma cells, according to Wang et al. [64].

### 3.3. Baicalein Induces Apoptosis in Cancer

Apoptosis, also known as programmed cell death, is disrupted in malignant cells, resulting in an imbalance between cell growth and apoptosis [65]. The biological events that occur in apoptosis have been studied extensively. However, it is generally recognized that the cell’s characteristics change because of these successive occurrences and that the cell eventually dies. As a result, controlling apoptosis is a crucial technique for controlling cancer growth. Baicalein has been found to induce apoptotic cell death in malignant cells of various kinds. It triggers apoptosis via both mitochondrial (intrinsic) and receptor-conciliated (extrinsic) mechanisms [66].

The anti-malignancy impacts of Baicalein are thought to be because of the transfiguration of many signaling axes, as well as the PI3K/AKT, which inhibits cellular growth while eliciting apoptosis, stimulation of the caspase cascade, and the intrinsic (mitochondrial) apoptotic trail [67,68], and DNA breakdown in tumor tissues [69,70]. The PI3K/AKT signaling tract is a condemnatory controller of apoptosis and performs a crucial role in the cell growth of mammals, distinction, autophagy, and longevity [71]. Activating or impeding the PI3K/AKT signaling axis has been shown to control cancerous cell viability in vitro and mutagenicity, invasion, and migration in vivo [72,73]. In several human cancers (breast cancer cells [74], renal carcinoma cells [70], glioma [71], cervical cancer cells [75], human epidermoid malignancies [76], and bladder carcinoma cells [77], etc.), baicalein-influenced apoptosis is controlled via suppression of the PI3K/AKT axis (Figure 3). After Baicalein treatment, the PI3K/Akt signaling axis repression of the PI3K/Akt signaling axis promoted apoptotic cell demise in MCF-7 and MDA-MB-231 [74]. It is interesting to note that the antagonist of kappa-B (IκB) is a critical controller of NF-κB signaling, and disruptions in this pathway have been linked to various malignancies [78,79]. Additionally, the Akt pathway plays a significant role in tumor development [80]. Baicalein administration can counteract these effects by reducing the concentrations of p-Akt, p-mTOR, NF-κB, and p-IκB while increasing IκB expression [74]. Studies have also shown that Baicalein enhances the activity of Bax, a pro-apoptotic protein, leading to a depletion in the anti-apoptotic protein Bcl-2 [81].

Apoptosis can be triggered through mitochondrial (intrinsic) and receptor-mediated (extrinsic) mechanisms, and Baicalein has been found to modify elements of both pathways [66]. The Bcl-2 family of proteins are mechanically similar proteins involved in the intrinsic apoptotic process. This family includes both pro-apoptotic (Bax, Bad, Bak, etc.) and anti-apoptotic (Bcl-2, Bcl-xL, mcl-1, Bcl-w, etc.) proteins, with the balance of these proteins determining cell fate. For example, in human tongue cancer cell lines (SCC-4), Baicalein was found to effectively induce apoptosis by increasing the concentrations of pro-apoptotic elements (Bax) and decreasing the concentrations of anti-apoptotic protein Bcl-2 [48]. Increased levels of pro-apoptotic to anti-apoptotic Bcl-2 proteins can induce the release of cytochrome C and other apoptotic agents from the mitochondria to the cytoplasm, which activates the caspase cascade and eventually triggers apoptosis in the relevant tissues [82]. Baicalein has been found to raise protein mRNA concentrations of death receptor 5 (DR5) and other TRAIL (TNF-related apoptosis-inducing ligand) receptors, which are part of the extrinsic apoptotic cascade, in malignant cells compared to ordinary cells. This suggests it could potentially be used as a chemotherapeutic agent [49]. Another study found that Baicalein caused mitochondrial malfunction and death in a human liver cancer cell line (HepG2) by downregulating Bcl-2 expression [83]. Caspase cascades play an important role in programmed cell death, particularly the stimulation of death protease (caspase 3) activity [84]. Baicalein has been shown to up-regulate caspase-3 activity in various cancers, including bladder carcinoma, lymphoma, osteosarcoma, and pancreatic cancer [85].

Baicalein affects the apoptotic pathway through the production of ROS, which was discussed in Section 3.1. ROS is mainly involved in normal metabolic functions but can also contribute to the development of disease processes [86]. High levels of ROS can lead to oxidative stress and mitochondrial deregulation [45]. Because of this, ROS generation is a technique that non-surgical cancer therapies, such as chemotherapy, radiation, and photodynamic treatment, can use [87]. Based on its metabolic activities and intensity, Baicalein can act as an antioxidant and pro-oxidant. Baicalein-influenced ROS can cause endoplasmic reticulum stress and eventually stimulate the Ca^2+^-mediated mitochondrial death cascade of apoptosis [47,48]. Baicalein also increases the production of BNIP3 (Bcl-2/adenovirus E1B 19 kDa protein-interacting protein 3), which is a protein stimulated by ROS and promotes apoptosis [88]. Moreover, baicalein-triggered ROS enhances DR5 expression in human prostate carcinoma cells, initiating the extrinsic apoptotic pathway [49].

Anti-cancer medications frequently aim at the p38 MAPK pathway, which initiates and causes apoptosis by activating various caspases [89]. In lung, bladder, and breast malignant tissues, Baicalein increased p38-MAPK phosphorylation and diminished the protein concentration of survivin, a component of the anti-apoptotic gene group [90]. With the help of ERK/p38 and MAPK axis, both Baicalein and Baicalin enhanced apoptosis by initiating caspase-3 and caspase-9 activity, suppressing Bcl-2, and raising Bax or p53 [91]. 

In addition to all of the mechanisms described, the inhibition of 12-LOX (Platelet-type 12-Lipoxygenase) has been identified as a potential approach for cancer prevention [92]. Some research has mentioned that Baicalein is a 12-LOX inhibitor. Baicalein reduced levels of the anti-apoptotic proteins Bcl-2 and Mcl-1 while increasing concentrations of the pro-apoptotic agent Bax by inactivating 12-LOX. This produced apoptosis by causing the ransom of cytochrome c from mitochondria into the cytoplasmic environment, stimulation of caspase-9, caspase-7, and caspase-3, and eruption of the caspase-3 precursor poly ADP-ribose polymerase (PARP) [93].

Moreover, it has been found that Baicalein modulates the degrees of the PI3K/Akt axis by inhibiting the manifestation of lncRNA (long non-coding RNA) in cervical malignancy [94]. This research suggests that Baicalein can generate anti-cancer actions, including apoptosis, by regulating short and lncRNA levels. Baicalein has been demonstrated to up-regulate lncRNA PAX8-AS1, which interacts with miR-17-5p and induces apoptosis in breast carcinoma cell lines in vitro [95]. Baicalein triggers apoptosis by down-regulating mir-106 expression in T24 cancerous bladder cells, which results in decreased phosphorylation of c-JNK (c-Jun N-terminal kinases), ERK, and MEK [96]. Baicalein activates miR-183 and promotes apoptosis in certain osteosarcoma cell populations (MG-63 and Saos-2) by down-regulating ezrin expression [97]. In HeLa cells (cervical carcinoma cell line), Baicalein may also target the Wnt/-catenin signaling cascade to induce apoptosis by limiting catenin nuclear translocation and decreasing Wnt activity, most likely via CCND1 [98]. In a preclinical study using a mouse xenograft T-lymphoblastic leukemia model 143B, Baicalin treatment targeted catenin and its relevant genetic elements, namely cyclin-D and c-myc, and down-regulated those that caused apoptosis in various cancer cells like osteocarcoma MG-63 and Jurkat cells [99,100,101].

### 3.4. Suppression of Cancer Stem Cells by Baicalein

Cancer stem cells (CSCs) or tumor-inducing cells (TICs) are composed of a massive number of cancer cells with the ability to restart, which can spread colonies and initiate tumor cell growth [102]. Some cell surface markers are used to determine CSCs, for instance, CD44, CD24, and CD133 [103]. Evidence suggests that tumorigenesis and metastasis originated from CSCs. Baicalein is a promising anti-cancer treatment strategy because it can suppress CSCs with comparatively fewer side effects [104].

**Figure 4:**
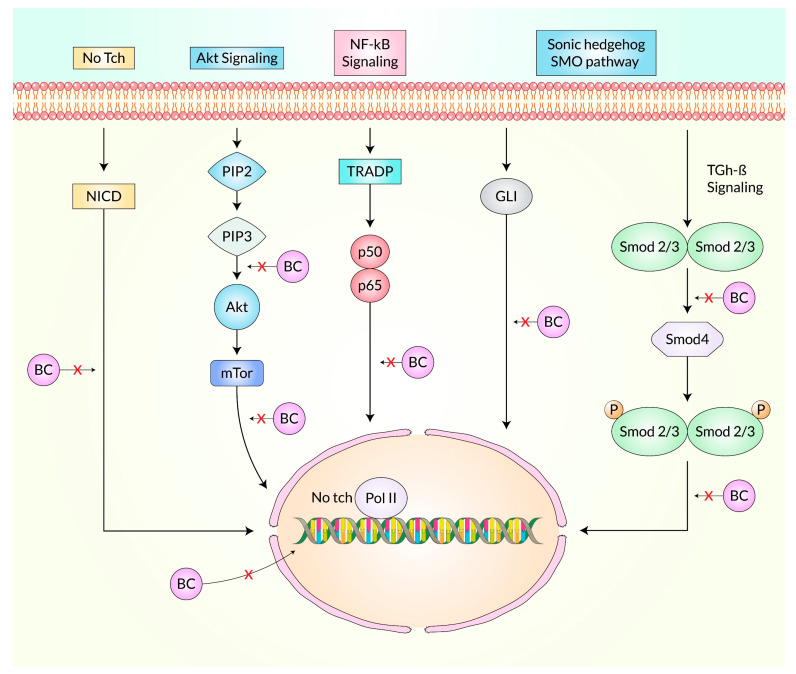
Effect of Baicalein on different signaling pathways. The schematic view represents the molecular mechanisms of Baicalein inhibiting cell proliferation through the Notch/Hes pathway. Cells undergo apoptosis following the treatment of Baicalein, which hampers the signal transduction between Akt and NF-κB signaling pathways. CSCs lose their self-replicating feature after Baicalein treatment, downregulating the Shh receptor and effectors.

Baicalein is a potential therapeutic option and targets different molecular routes to display anti-cancerous properties (Figure 4). Breast-, cervical-, and thyroid-based CSCs lose their proliferation ability and undergo apoptosis after treatment with Baicalein because it targets phosphoinositide 3-kinase (PI3K)/Akt and the signal transducer and activator of transcription 3 (STAT3) axis [105]. It also down-regulates Notch 1/Hairy and enhancer of the split (Hes) pathway (Figure 4) and diminishes cancer cell proliferation [75]. Moreover, it regulates various functions in gastric, prostate, and thyroid CSC lines via the mammalian target of rapamycin (mTOR) pathway, blocking cell growth and metastasis and the progression of cancer cell apoptosis and autophagy [55]. Baicalein reduced the generation of nuclear factor kappa B (NF-κB) in cervical, ovarian, hepatocellular, and lung CSCs [106]. In addition, it can target the Sonic Hedgehog (SHH) pathway and suppresses the self-replicating properties in CSCs [107]. In a recent study, Baicalein significantly reduced the presence of Gli-2, a crucial transcription factor in the SHH pathway, in the nucleus. Silencing Gli-2 expression in pancreatic CSCs counteracted the inhibitory effects of baicalein and reduced baicalein-induced apoptosis [107].

MDA-MB-231/IR (breast cancer cell populace) cells have stem-cell-like properties. When treated with Baicalein, the expression level of interferon-induced protein with tetratricopeptide repeats 2 (IFIT2) is increased. The expression rate of the stem cell markers (Oct3/4 and ABCG2) is reduced after treatment with Baicalein. The Western blot assay confirmed the results indicating the apoptosis of MDA-MB-231/IR cells [103]. One study reported that Baicalein inhibits SiHa and HeLa (cervical cancer cell populace) cell proliferation and reduces the rate of P-Akt and phosphorylated glycogen synthesis kinase 3 beta (P-GSK3b) [108]. Additionally, Baicalein represses the function of CSCs in human NSCLC xenografts via the Src/Id1 pathway and minimizes cancer cell metastasis [109].

Baicalein inhibited the growth of prostate cancer cells by downregulating the androgen receptor (AR) through AR-N-C dimerization and AR-coactivator interaction. This is significant, because the AR receptor accelerates prostate cancer growth and metastasis [110]. Baicalein has also been shown to downregulate various cell proliferative markers and suppress CSCs in multiple types of cancers, such as gallbladder carcinoma, glioma, lymphoma, leukemia, nasopharyngeal carcinoma, thyroid carcinoma, and osteosarcoma [109].

### 3.5. Cell Cycle Arrest Induction by Baicalein in Cancer

A cell cycle is a process through which cells are produced, matured, and divided in all living organisms [111]. The cell cycle of mammalian species has four distinct non-overlapping stages, including gap-1 or G1 phase, synthesis or S phase, gap2 or G2 phase, and mitosis or M phase [112,113]. In the case of cancer cell division, the cell cycle plays a significant role because its dysregulation may result in uncontrolled and abnormal cell proliferation.

The cell cycle is regulated by multiple genes, including three primary types: cyclin, CDK, and CDK inhibitor (CKI) [114,115]. Cyclin constituents and many CDKs, including CDK4/6-cyclin D and CDK2-cyclin E, play a crucial role in regulating the progression of the cell cycle. The cytokines and CKIs both positively and negatively control CDKs, which, in turn, accelerates cell proliferation [14]. Targeting the cell cycle has shown to be an effective therapeutic strategy for treating cancer cells.

Recent studies have shown that the Baicalein compound can induce apoptosis and can suppress cancer cell progression by blocking the cell cycle at different stages [81,116,117,118]. For example, cell cycle development and multiplication of prostate cancer cells were suppressed by Baicalein therapy [119,119,120]. Baicalein inhibited cell proliferation by arresting growth at the G1 and S phases of the cell cycle in prostate cancer cells. Besides this, Baicalein can inhibit the production of cyclins and CDKs and up-regulate CKI expression to prevent the abnormal accumulation of tumor or cancer cells [121,122]. Baicalein delayed cancer cell development in prostate cancer cell lines, such as LNCaP, PC3, and DU145, by preventing the release and activation of growth-stimulating molecules, including CDK inhibitors like p21 or p27 [123,124,125]. It can regulate cell cycle mediators by increasing the expression of Rb, p53, p21, and p27 and decreasing the expression of cyclin D1, cyclin E, CDK4, and phosphor-Rb [126].

Furthermore, after being treated with Baicalein, several malignancies have shown significant reductions in protein levels, such as CDK2, CDK4, and cyclin E2 [117,127]. Multiple studies showed that the cell cycle was significantly halted in the G1/S phase, followed by a reduction in the G2/M stage [128,129,130]. Baicalein inhibits the growth of human cancer cells by stopping the cell cycle and causing cancer cells to die [28,60], but further study and clinical trials are needed to establish this statement.

## 4. Cell-Signaling Molecular Mechanisms of Baicalein for Cancer Treatment

The anticancer effects of Baicalein are mediated by interacting with specific signaling pathways. The key pathways include ERK/MAPK (extracellular-signal-regulated kinase/mitogen-activated protein kinase), mTOR (mammalian target of rapamycin), PI3K/Akt/NF-κB signaling axis, PARP (poly ADP-ribose polymerase), MMP-2 (matrix metalloproteinases-2), MMP-9 (matrix metalloproteinases-9), caspase-3/-8/-9, Wnt/β-catenin, and Smad4 (suppressor of mothers against decapentaplegic 4) by mediating the TGF-β signaling pathway, Notch-1/Hes, Nrf2 (nuclear factor erythroid 2-related factor 2)/Keap-1 (Kelch-like ECH-associated protein-1), AMPK (adenosine monophosphate-activated protein kinase), miR 183/ezrin, Src/Id1, ROS cascade, and SHH [74,88,100,131,132,133,134].

Baicalein has been found to induce apoptosis in malignant cells through both intrinsic and extrinsic pathways by releasing cytochrome-C from mitochondria. It activates the TNFR-associated death domain (TRADD) via the extrinsic pathway. It also triggers autophagy in malignant cells and interferes with the generation of autophagosomes at multiple stages by engaging the AMPK/ULK1 and PI3K/Akt/mTOR signaling axes [135]. The ERK/p38 MAPK signaling pathway is believed to down-regulate the anti-apoptotic element Bcl-2 and activate caspase-3 and caspase-9 in cancerous cells. An in vitro study demonstrated that Baicalein treatment causes time-dependent increases in the phosphorylation of ERK1/2 and AMPKα in H1650 and A549 cells.

Baicalein reduces cancer spread and induces death in malignant cells by affecting various steps of the metastatic tumor process, including invasion, survival, arrest, and colonization. Additionally, Baicalein has been found to induce autophagy in various types of cancer cells. This is demonstrated by the fission of LC3, measurement of autophagic degression activity (Flux), autophagosome creation, stimulation of Atg7/Atg5, vacuolar protein sorting 34, and Beclin-1 participation in the AKT/mTOR axis, as well as the activation of RelB/p52 proteins [55,136]. In addition to apoptosis and autophagy, this compound also causes cell cycle arrest at particular checkpoints in cancerous cells. Moreover, several research studies showed that Baicalein possesses strong topoisomerase inhibition activity by stabilizing topoisomerase I-DNA cleavage complexes, which subsequently inhibited the religation step. The capacity of Baicalein to stabilize the covalent topoisomerase I-DNA complex in both in vitro and in vivo settings is nearly equivalent to that of the established potent topoisomerase I inhibitor, camptothecin (CPT) [137,138,139]. The role of Baicalein on diverse malignant cells is summarized in Table 1, focusing on the signaling mechanism. The data presented in Table 1 are derived from various pre-clinical and in vitro studies that examined the effects of Baicalein on cancer cells.

### Akt Is the Principal Target of Baicalein Following PI3K/Akt, mTOR, MAPK, PI3K/FoxO, and NF-κB Signaling Pathway in Cancer

‘Akt’ is the set of enzymes involved in cell development and survival processes. These enzymes help transmit impulses within cells. The Akt enzyme is a variation of serine/threonine-protein kinase. Since its discovery as a proto-oncogene, it has become a pivotal target of consideration because of its remarkable action in maintaining multidimensional cytological activities, including development, proliferation, metabolism, survival, apoptosis, transcription, and protein synthesis. Tyrosine kinases, integrins, B- and T-cell receptors, cytokine receptors, G-protein-coupled receptors, and other stimulating agents trigger the Akt signalizing pathway, which causes phosphoinositide 3-kinase (PI3K) to produce phosphatidylinositol triphosphates (PIP3) [157,158].

The Akt pathway, specifically the PI3K/Akt pathway, must be strictly controlled because it has numerous downstream consequences, such as cell growth and proliferation, cell survival, glucose metabolism, protein synthesis, and transcriptional regulation when activated [159,160]. Decreasing PIP3 concentrations is one of the methods by which the process is adversely controlled. PIP3 is changed by phosphatase and tensin homolog (PTEN) into PIP2 (phosphatidylinositol diphosphate), which inhibits PI3K [161]. Akt is frequently overactivated in cancer cells due to loss of PTEN activity (PTEN acts as a tumor suppressor). The PI3K-Akt axis controls PTEN concentration by modifying its transcription and action. Peroxisome proliferator-activated receptor delta (PPARδ) and tumor necrosis factor (TNF) are controlled by Akt-activated transcription factor NF-κB, which, in response, suppresses PTEN synthesis [105].

**Figure 5:**
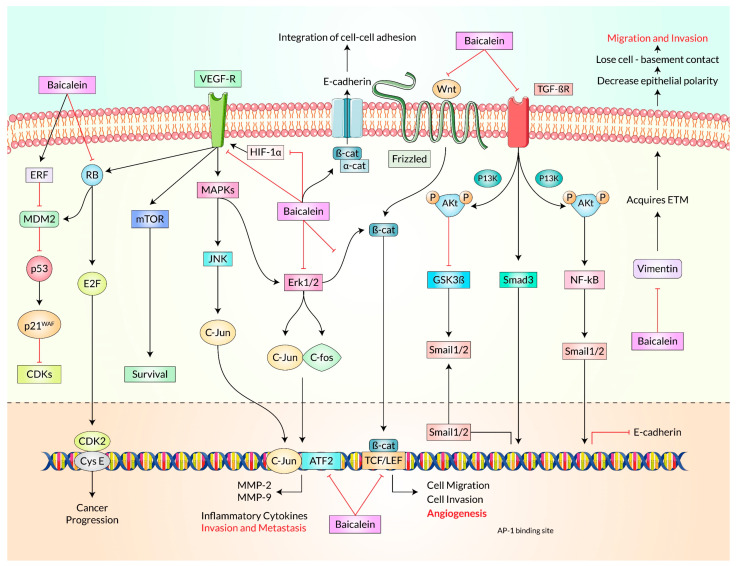
Mechanisms of Baicalein in regulating the cancer signaling pathways.

The PI3K pathway promotes NEDD4-1, an E3 ligase recognizing PTEN for destruction. As a result, PTEN is further suppressed in a positive feedback process if Akt is active [73]. Akt controls cell proliferation by affecting the tuberous sclerosis complex (TSC1/TSC2) and mTORC. Akt helps in cell growth by activating the CDK blockers p21 and p27. Akt is a major modulator of cell viability, exerting inhibitory effects on pro-apoptotic factors such as Bad and blocking the production of pro-apoptotic kinases by transcriptional mediators like FoxO. In addition, Akt plays a significant role in maintaining metabolism by phosphorylating proteins such as AS160 and PFKFB2. The proteins vimentin and paladin play crucial roles in maintaining cellular stability and regulating cell morphology and attachment. Vimentin, belonging to the intermediate filament family of proteins, provides the necessary resilience to cellular stress. At the same time, paladin is a component of actin-containing microfilaments that control cell attachment, contraction, and morphology. Akt phosphorylates vimentin and paladin during epithelial-to-mesenchymal transition (EMT) to aid cell infiltration and mobilization. Additionally, Akt modulates the NF-κB signaling pathway by phosphorylating IkappaB kinase (IKK) and tumor progression locus 2 (Tpl2) (Figure 5) [105,157,158,161]. Baicalein has been found to target the PI3K/Akt and STAT3 (Signal transducer and activator of transcription 3) pathways in breast, cervical, and thyroid malignancy, which are important for cancer cell survival and growth. It suppressed the proliferation while persuading apoptotic cell demise and autophagy in certain cancerous cell phenotypes in vitro and in vivo [94,95,132,162]. Additionally, it altered the Akt/mTOR signaling activity in gastric, prostate, and thyroid malignant cells, preventing them from growing and metastasizing while inducing autophagy and cell death [74,143,163].

Baicalein drastically reduced the expression of the lncRNA, BDLNR, ultimately preventing the survival, proliferation, and migration of cervical tumors. Moreover, Baicalein also prevented tumor formation in animal models by downregulating the PI3K/Akt cascade [162]. Baicalein therapy has also been found to induce apoptosis in cell populations from breast malignancy, such as MDA-MB-231 and MCF-7, by blocking the PI3K/Akt signaling axis [164].

It is widely acknowledged that the NF-κB and Akt/mTOR networks critically control the cell’s autophagic activities. Surprisingly, Baicalein administration significantly decreased the concentrations of p-Akt, NF-κB, p-mTOR, and p-IκB and increased the expression of I-κB. According to reports, by inhibiting the Akt/mTOR and NF-κB pathways, Baicalein increased the cisplatin responsivity and initiated autophagy in gastric carcinoma cells [22,165]. It was also demonstrated that Baicalein activates the Akt, which causes autophagy in human T24 cell lines of bladder malignancy [74]. Another study found that Baicalein boosted LC3B and BECN1 while decreasing the expression of critical autophagy-related molecules, namely p-Akt, Akt, p-mTOR, mTOR, NF-κB, and p-IκB in MDA-MB-231 and MCF-7 [166,167]. Multiple studies have noted the importance of Baicalein in preventing EMT (epithelial–mesenchymal transition), and that evidence suggests new ideas for decreasing metastatic techniques [55,168,169,170]. In addition, Baicalein has been shown to prevent EMT and reduce metastatic processes by suppressing the PI3K/Akt/NF-κB signals, inducing the apoptotic mechanism, and causing EMT transformation into MET [134]. Baicalein therapy has also been found to prevent metastasis by blocking the Akt, mTOR, and caveolin-1 pathways in malignant prostate cells (DU145 and PC-3) [171] and inhibiting the PTEN/Akt/HIF-1 signaling cascade in AGS cells, which restored hypoxia-induced 5-FU resistance [172].

**Figure 6:**
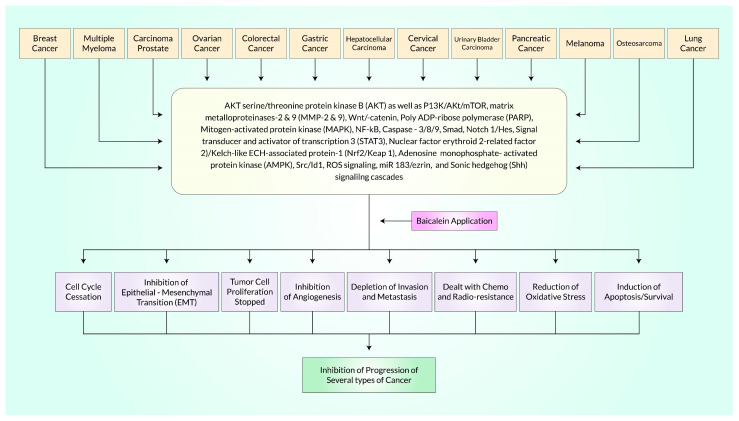
Baicalein can limit the growth of various malignancies by binding to and interacting with many different molecular targets. These cellular points, which play a role in preventing multiple malignancies, are presented here. This figure was updated and modified after Liu et al. [30].

Baicalein has been extensively studied in pre-clinical research. It has been found to inhibit the expression of several proteins, including MMP-2/9, CDK, Akt, and MAPK, which are important for the development and progression of cancer [73,162,173,174,175,176,177,178]. Baicalein has also been shown to inhibit cancer cell migration by suppressing the Wnt/-catenin and MAPK pathways [148]. Additionally, Baicalein promoted autophagy by modulating the cascade of ROS and down-regulating Beclin 1, Vps34 (Vacuolar protein sorting 34), Atg5, and Atg7 [106]. Dysregulation of the 5′AMPK/ULK1 (Unc-51-like autophagy activating kinase) connection and repression of mTORC1 were also linked to the initiation of autophagy [47]. Baicalein induces apoptosis by suppressing the expression of Bcl-2, IAP, and XIAP while increasing the level of p53, caspases, Bax, and cleavage of PARP [20,48,50,94,132,164,179,180]. NF-κB (Nuclear factor kappa B) expression was also negatively regulated by Baicalein in thyroid, ovary, lung, cervical, and hepatocellular malignancies (Figure 6) [100,131,181,182].

The impressive therapeutic properties of Baicalein, such as its angiogenesis inhibition, inflammatory process blocking, invasion prevention, counter-metastasis, and antioxidant, contra-proliferative, and apoptotic activities, have been demonstrated by numerous investigations by targeting multiple pathways, including ERK/MAPK, Wnt/-catenin, and PI3K/Akt/NF-κB [74,88,100]. Baicalein has also been shown to inhibit breast cancer cell MDA-MB-231 migration and invasion by down-regulating the expression of SATB1, Wnt/-catenin, and MAPK [183,184].

**Figure 7:**
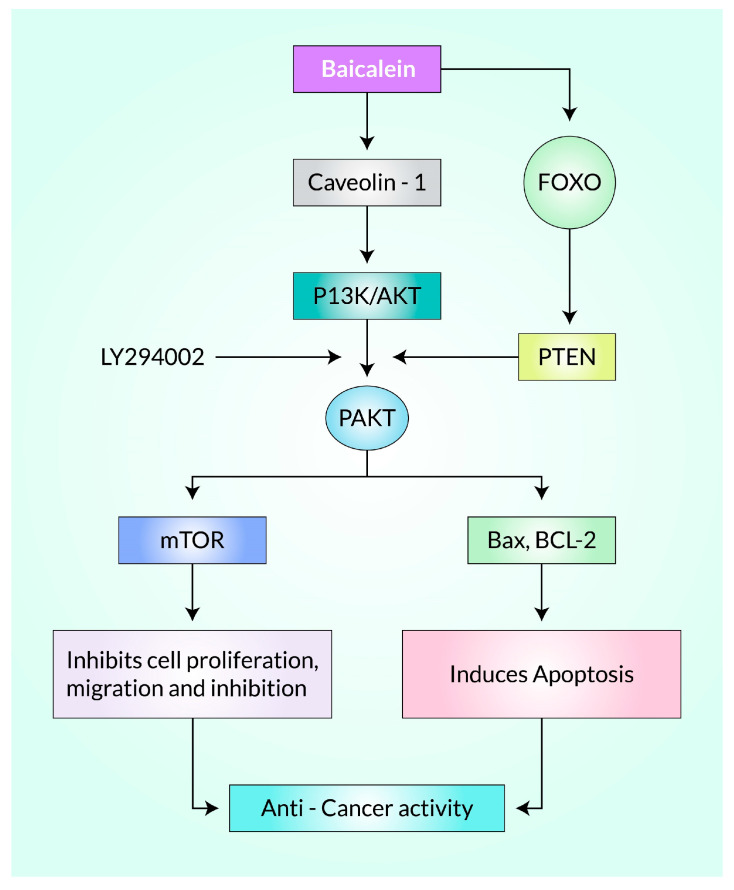
Baicalein inhibits cancer cell progression by targeting P13K/FOXO pathways.

There is another pathway by which Baicalein may show anticancer activity (Figure 7). FoxOs (Forkhead Box Class O proteins) are transcriptional factors, which include FoxO1/FKHR, FoxO3/FKHRL1, FoxO4/AFX, and FoxO6, that play a significant role in cancer development and migration. They regulate growth, apoptosis, metastasis, metabolic functions, senescence, and the cancer microenvironment through various modifications, such as phosphorylation, ubiquitination, acetylation, and methylation. Although it is now known that the Akt/PI3K signaling cascade is primarily responsible for controlling FoxOs, its specific function in the development and spread of cancer is still not fully understood [185,186].

In NSCLC (Non-small cell lung cancer) cells, Baicalein therapy activates AMPKα/MEK/ERK1/2 signaling cascades by triggering phosphorylation, which cross-talks between FOXO3a and RUNX3 (RUNX family transcription factor 3) [25]. Additionally, Baicalein treatment reduces the expression of Caveolin-1 (cav-1) in prostate cancer cells, DU145, and PC-3 cells, resulting in the downregulation of AKT and mTOR levels. Diminishing Cav-1 expression can reduce the metastatic effect of prostate cancer. Moreover, Baicalein accelerates the expression of FoxO3a protein, which is a crucial factor in regulating P13K/AKT signaling pathways through Phosphate and tensin homolog (PTEN) and has anti-tumor activity as a subclass of FoxO proteins. Inhibition of the PI3K pathway by PTEN further reduces the mobility of prostate cancer cells (Figure 7) [149].

## 5. Baicalein-Based Drug Design for Cancer Treatment: A Focus on Nanomedicine Development

Baicalein is a natural compound with potential anti-tumor effects. However, its application has been hindered due to poor solubility, low bioavailability, and short half-life. To address these problems, nanotechnology has been proposed as an alternative solution. Several studies have been conducted to investigate effective delivery systems for Baicalein using different methods, which are discussed below.

Baicalein-loaded nanoliposomes were designed by the film hydration method to improve their bioavailability and anti-tumor effects. The results demonstrated that Baicalein–Liposome (BAI-LP) exhibited more than 1.5 times higher tumor inhibition (66.34 ± 15.33%) compared to the free form of Baicalein (41.89 ± 10.50%) in a mouse model carrying U14 cervical tumor [187]. Even though BAI-LP showed a better result, this finding alone does not definitively establish BAI-LP as an effective delivery system. Further evaluation of BAI-LP is necessary to determine its efficacy, including testing at different combinations and concentrations to be a viable delivery system for Baicalein in cancer treatment.

However, the ultrasonic-film hydration method was used to develop glycyrrhizic acid–Baicalein (GA-BAI) nano-micelles that exhibited excellent water solubility, with an increase of 4600-fold. This result suggests that this system could be a novel method for improving the solubility of Baicalein [188]. In another study, long-circulating nanoliposomes loaded with Baicalein were developed using the dimethyl ether injection process. The results showed a 4.52-fold enhanced oral bioavailability compared to free Baicalein solution in pharmacokinetic studies of mouse plasma [189]. Another study utilized the nanoprecipitation method to formulate polymeric nanocapsules loaded with Baicalein, demonstrating a 216-fold increase in the anti-cancer effects for MCF-7 cells and a 31-fold increase for MDA-MB-231 after 1 day of prolonged incubation. This suggests that the system enhanced the anti-cancer effect by increasing the ROS level and promoting a mitochondrial-dependent apoptosis mechanism [190].

In a recent study, Baicalein (15–16 µg/mL) was encapsulated into chitosan (CS) nanoparticles and cross-linked with cinnamaldehyde—an anti-cancer agent—using the emulsion cross-linking method, resulting in uniform nanoparticles. The cytotoxicity of these CS nanoparticles loaded with Baicalein was tested in MCF7 cells, and a significant increase in the inhibition rate was observed. This indicates the potential of this approach for improving the anti-cancer effect of Baicalein in vitro [191]. A study aimed to evaluate the anti-tumor efficacy of hyaluronic acid, Baicalein (BAI), and Doxorubicin (DOX) (HA-BAI/DOX)-loaded nanostructured lipid carriers in both MCF-7/ADR cells and mice bearing MCF-7/ADR breast cancer models. The results indicated that the tumor inhibition rate of the HA-BAI/DOX solution was 88%, demonstrating the system’s excellent anti-tumor effect in vitro and in vivo. Furthermore, this system achieved targeted co-delivery of BAI and DOX, while minimizing systemic toxicity [192].

A multifunctional Paclitaxel–Baicalein (PTX/BAI) nanoparticle was synthesized using the nano-precipitation method to investigate its effectiveness against multidrug-resistant cancers. This system exhibited better anti-tumor activity and increased synergistic anti-cancer effect in A549. The nano-emulsions (NEs) co-encapsulating Paclitaxel and Baicalein by high-pressure homogenization were prepared to overcome multidrug resistance (MDR) in breast cancer. This investigation demonstrated that PTX/BAI NE could increase intracellular ROS levels, reduce cellular glutathione (GSH) levels, and trigger caspase-3 dynamism in MCF-7/Tax cells. Moreover, it exhibited higher efficacy in inhibiting tumors in vivo [193].

Using the emulsification method, researchers designed Transferrin-equipped Docetaxel and Baicalein-loaded solid lipid nanoparticles (Tf-D/BAI-SLNs), observing a 90% encapsulation efficiency and pronounced synergistic effects with low systematic toxicity on A549/DTX, indicating the potential for lung cancer treatment [26]. Baicalein-loaded nano-emulsions were also found to enhance oral bioavailability with an encapsulation efficiency of 99.31% and higher cellular uptake than free Baicalein, indicating potential as a facilitating and transcellular vehicle [194].

In a recent study, researchers used a high-pressure homogenization method to create baicalein–nicotinamide (BAI-NCT) nanocrystals. The results showed that these nanocrystals increased the dissolution rate of baicalein by 2.17 and 2.54 times compared to coarse powder when tested in FaSSIF-V2 and FaSSGF, respectively.

A recent study synthesized baicalein–nicotinamide (BAI-NCT) nanocrystals using a high-pressure homogenization method. This study demonstrated that the synthesized nanocatalyst depicted a 2.17- and 2.54-fold higher dissolution rate than coarse powder of baicalein in FaSSIF-V2 and FaSSGF, respectively. This approach is considered a promising strategy for improving the dissolution rate and bioavailability of hydrophilic natural products like baicalein [195]. In another study, the researchers evaluated baicalein-loaded cinnamon oil solubility and oral bioavailability (BAI-CN). The results showed that BAI-CN enhanced anti-cancer activity by 19- and 23-fold after 12 and 24 h of treatment, respectively, compared to baicalein alone. Additionally, BAI-CN was an effective anti-cancer agent against the MDA-MB-231 breast cancer cell line [196]. The challenges of poor solubility, low bioavailability, and short half-life of Baicalein were improved at varying folds by implementing nanotechnology-based solutions such as nanoliposomes, nano-micelles, polymeric nanocapsules, chitosan nanoparticles, and nanoprecipitation methods to enhance the anti-cancer effects of Baicalein.

## 6. Conclusions and Future Perspectives

In conclusion, despite the increase in cancers and the limited treatment options available, natural products like Baicalein have gained popularity as a safer alternative to conventional treatments. However, the effectiveness of this compound needs to be extensively evaluated and validated before considering it as a treatment option. Therefore, this study assessed existing research findings and highlighted the numerous anti-cancer properties of Baicalein and the molecular pathways established so far for the purported benefit of Baicalein as an anticancer drug.

Baicalein possesses various mechanisms for inhibiting cancer cells, including through the MAPK pathway, inactivating 12-lipoxygenase, blocking PI3K/Akt, down-regulating anti-apoptotic protein, up-regulating tumor suppressor p53, decreasing oncogene levels, and inducing tumor suppressor gene production. Numerous in vitro and pre-clinical studies showed that Baicalein has anti-cancer properties that suppress cell growth and differentiation by blocking the cell cycle, inhibiting metastasis, accelerating apoptosis, and elevating autophagy via various signaling pathways. Thus, Baicalein could be considered an emerging anticancer drug that would be safer and cost-effective since it can be extracted from natural biomass.

However, despite the promising anti-cancer properties of Baicalein, its therapeutic applicability has been limited by low bioavailability and poor pharmacokinetics. To overcome these limitations, future research should focus on increasing Baicalein’s bioavailability using system biology, RNA sequencing, proteomics, genomics, bioinformatics, and nano-medicine-based applications, making them more desirable for developing novel anti-cancer medications. Therefore, further substantiation-based solo clinical trials and greater synergistic approaches are also needed to confirm the effectiveness and safety of Baicalein as a possible therapeutic agent for various cancerous diseases. Therefore, enhancing bioavailability and pharmacokinetics will be crucial in the future development of Baicalein as an emerging anticancer drug.

## Figures and Tables

**Table 1:** Effects of Baicalein on cancer cells.

In Vivo								
Compound	Experimental Model	Dose	Treatment Duration	Standard/ Control	Targeted Pathways	Mechanism of Actions	Outcomes	References
Baicalein	Mice pulmonary carcinogenesis model	12 mg/kg body weight	16 weeks	Mice that received corn oil during the research period.	ROS-induced mitochondrial DNA damage by free radical scavenging potential	↓Mitochondrial ROS Production ↓Mitochondrial swelling, ↑VDAC expression, ↑Activity of Krebs Cycle enzymes, ↑Activity of METC enzymes	Inhibited lung carcinogenesis in mice	[136]
Baicalein	Human breast carcinoma MCF-7 and MDA-MB-231 cells xenograft in female BALB/c nude mice	100 mg/kg body weight	21 days	Untreated nude mice that received in-vitro-cultured MCF-7 and MDA-MB-231 cells into the second left breast pad via subcutaneous injection.	PI3K/AKT/mTOR Pathway	↓Manifestation of p-AKT, p-mTOR, NF-κB, and p-IκB, ↑Manifestation of IκB at the protein level, ↓Proportion of p-AKT/AKT and p-mTOR/mTOR, ↓Bax/BCL-2 ratio	Induced apoptotic cell demise and autophagy by blocking cell multiplication in breast cancer cells. Significantly reduced cell progression and metastasis.	[74]
Baicalein	BON1 (Pancreatic neuroendocrine tumor cell line) cells xenograft in female nude mice	10 mg/kg body weight	7 weeks	Untreated female nude mice that received BON1 cells into the head of the pancreas.	Mitochondrial pathway, cleavage of caspase 3	↓Expression of Survivin in BON1 Cells, ↓Bcl-2, ↑Bax, ↓MMP-2, ↑MMP-9	Induced apoptosis, inhibited cell invasion and migration, and reduced tumor volume.	[140]
Baicalein	Human lung carcinoma A549 and NCI-H1299 cells xenograft in 24 SPF female nude mice	__	__	Mice were inoculated subcutaneously with A549 cells in the right axillary and treated with saline.	SMYD2/RPS7 signaling pathway	↓Expression of SMYD2	The carcinogenic effect of SMYD2 was blocked; thus, the growth, migration, and infiltration of human non-small cell lung cancer cells were stopped.	[141]
Baicalein	Human oral Cancer SCC25 cells heterograft in BALB/c nude lab rat	30 mg/kg body weight	21 days	Control group mice were treated with PBS (0.01% DMSO).	Sp1/NF-κB-dependent pathway	↓expression of transcription factor Sp1, ↓NF-κB, ↓p65 and p50, cessation of cellular maturation process at the G0/G1 level	Suppressed the development of OSCC (human mouth squamous cell carcinoma) cells, induced apoptosis.	[142]
Baicalein	Human cervical cancer HeLa, SiHa, ME-180, and Caski cell lines xenograft in female athymic BALB/c nude mice	10 mg/kg/day	28 days	Tumor-inoculated mice were treated with 0.1 mL DMSO (0.25%)	Stimulated *PIK3CA* manifestation and PI3K/Akt axis	↓long noncoding RNA (BDLNR) expression, ↑Akt phosphorylation	Diminished cell multiplication, enhanced malignant cell death, blocked migration, and in vivo tumor progression reduction of malignancy of the cervix.	[143]
Baicalein	Human osteosarcoma 143 B, MG63 and U2OS cell lines xenograft in female BALB/c nude mice	40 mg/kg body weight	14 days	Tumor-inoculated mice were treated with buffer solution (10% DMSO + 40% PEG300 + 5% Tween-80 + 45% saline).	lncRNA-NEF oriented Wnt/β-catenin signaling axis	↓lncRNA-NEF, ↓Wnt/β-catenin	Inhibited tumor growth, invasion, and metastasis.	[144]
Baicalein	Colorectal cancer cell xenograft mouse model	10 & 20 mg/kg body weight	21 days	Untreated tumor-inoculated mice.	TLR4/HIF-1α/VEGF signaling trail	↓HIF-1α and VEGF expressions, ↓NFκB phosphorylation, ↓VEGF, ↓CD31, ↓MMP-2	Inhibited colorectal cancer growth and angiogenesis. Reduced the metastatic potential.	[145]
Baicalein and Baicalin	Human colon cancer cell line HCT116 in humanized NOD-scid IL2Rγ^null^ (NSG) mouse xenograft model	50 mg/kg body weight	29 days	Tumor-inoculated mice were treated with intraperitoneally injected solvent.	MAPK/p38/ERK1/2 signaling axis	The tumor cell cycle seized in the S phase and decreased in the G0/G1 phase, ↑phosphorylation of ERK and p38, ↓ hTERT expression	Induced cell cycle cessation of the malignant cells, apoptosis, and senescence. Blocked cell cycle progression. It also inhibited colony formation and migration.	[146]
Baicalein	Human nasopharyngeal cancer CNE1 and CNE2 cell lines heterograft in BALB/C nude mice	1.0 mg/kg, 2.0 mg/kg, and 3.0 mg/kg	12 days	Tumor-inoculated mice were treated with intraperitoneally injected cisplatin DDP and saline.	Extrinsic and intrinsic apoptotic axis	↑caspase-3, ↑caspase-8, ↑p62, ↑Bcl-2, ↓p-ERK/ERK, ↓p-Akt/Akt, ↓Bcl-2/Bax, ↑Atg12, ↑Atg5	Inhibited the development and multiplication of malignant nasopharyngeal cells, modified the cell cycle, and induced apoptosis	[147]
Baicalein	Murine model T cell lymphoma (EL4) cells heterograft in C57BL/6 male mice	10 mg/kg body weight	3 days	Tumor-inoculated mice were left untreated.	ASK1/Cytochrome-C/Caspase-3 cascade	↓Gli-2, ↓Sox-2, ↓SHH, ↓SMO, ↓Oct-4	Diminished the recurrence of cancer stem cells, Induced apoptosis	[148]
In Vitro								
	Human prostatic carcinoma PC-3 and DU145 cell lines	20 and 40 μM	24, 48, and 72 h	Untreated PC-3 and DU145 cells	Caveolin-1/PI3K/AKT/mTOR pathway	Inhibition of Cav-1/PI3K/AKT/mTOR pathway, ↑Bax expression, ↓Bcl-2 expression	Triggered apoptosis in androgen-free malignancy of prostatic cells by impeding their development. Also showed anti-metastatic properties.	[55]
Baicalin, Baicalein, and Wogonin	Human pancreatic carcinoma BxPC-3, HPAF-II, Capan-2, AsPc-1, MIA PaCa-2, and Panc-1 cell lines	0, 5, 15, and 50 μM	24 and 48 h	Untreated BxPC-3, HPAF-II, Capan-2, AsPc-1, MIA PaCa-2, and Panc-1 cells	Cleavage of caspase-3, -7, and Poly-ADP ribose polymerase (PARP)	↓Bcl-2, ↓Mcl-1, ↓Bcl-xL	Diminished proliferation and triggered apoptosis of malignant pancreatic cells.	[149]
Baicalein	Human bladder transitional cell carcinoma (BTCC) T24 cells	0, 50, 100, 150, and 200 μM	24 h	Untreated T24 cells	c-JNK and MEK/ERK trails, regulation of miR-106	↓miR-106, ↑p16, ↑p21, ↓cyclinD1, ↓Bcl-2, ↑Bax, ↓MMP-2, ↑MMP-9	Repressed proliferation and spread and caused apoptosis.	[96]
Baicalein	Human osteogenic sarcoma MG-63 and Saos-2 cell populace	0, 25, 50, 75, and 100 µM	24, 48, or 72 h	Untreated MG-63 and Saos-2 cells	miR-183/Ezrin pathway	↑miR-183, ↓Ezrin	Diminished cell proliferation, migration, and infiltration of osteosarcoma and induction of apoptosis in mentioned cancer	[97]
Baicalein	Human glioma cell line U251MG	80 μM	12, 24, 36, and 48 h	Untreated U251MG cells	AMPK Pathway	↑LC3II, ↓p-AMPK, ↓Caspase-3	Induced autophagy and apoptosis.	[150]
Baicalein	Human anaplastic thyroid carcinoma cells (FRO)	10 μM, 20 μM, 40 μM, and 80 μM	12, 24, 36, and 48 h	Untreated FRO cells	ERK/PI3K and Akt trail	↓Bcl-2/Bax, ↓p-ERK/ERK, ↑caspase-3, ↑caspase-8, ↑Bcl-2, ↑Atg5, ↑p62, ↑Atg12, ↓p-Akt/Akt	Induced apoptosis and autophagy, reduced cell colony formation, and arrested tumor cell cycles.	[73]
Baicalein	Human anaplastic thyroid carcinoma cells (FRO)	0, 10, 20, 50, and 100 μM	24, 48, and 72 h	Untreated FRO cells	ERK/p38, MAPK and Akt trail	↓Bax, ↓cytochrome-C, ↓PARP, ↓cleaved caspase-3, ↓Cox-2, ↑Bcl-2, ↑p-ERK, ↑Akt, ↓pJNK, ↑p38-MAPK	Induced apoptosis.	[151]
Baicalein	Human gastric cancer MGC-803 cell line	0, 5, 15, 25, 50 μmol/L	24, 48, 72 h	Untreated MGC-803 cells	PI3K/AKT signaling pathway	↑Lysosomal acid, ↑LC3, ↓p-AKT, ↑LC3-II/LC3-I, ↓p-PI3K, ↑p62	Induced autophagy.	[133]
Baicalein	Ovarian cancer HEY and A2780 cells	12.5, 25, and 50 μM	24 h	Untreated HEY and A2780 cells	Beclin 1 and ERK signaling pathway	↓Beclin 1, ↑LC3-II, ↑PARP, ↑p-ERK, ↑p-AKT	Induced autophagy, decreased cell viability	[152]
Baicalein	Human Ewing Sarcoma SK-ES-1 and RD-ES cell populace	5, 10, 20, 40, 80, and 160 μM	24, 36, and 48 h	Untreated SK-ES-1 and RD-ES cells	Mitochondrial apoptotic and death receptor pathway	↑Bax, ↓Bcl-2, ↑Bax/Bcl-2, ↑Cytochrome-C, ↑Caspase-3, ↑Caspase-9, ↑Caspase-8, ↑MMP-2, ↑MMP-9, ↑PARP	Inhibited Ewing’s Sarcoma cell viability and induced apoptosis.	[153]
Baicalein	Human Multiple Myeloma U266 cells	0, 20, 40, 80, and 160 μmol/L	0, 6, 12,24, and 48 h	Untreated U266 cells	Proteasomal degradation of IKZF1 and IKZF3	↓ IKZF3, ↑CRBN, ↓cIKZF1	Suppressed growth and promoted apoptosis of myeloma cells.	[154]
Baicalein	Malignant melanoma A375 and SK-MEL-28 cell lines	100, 50, 20, and 10 μM	24 h	Untreated A375 and SK-MEL-28 cells	Wnt/β-catenin or MEK/ERK signaling axis by regulating CCAT1 (Colon cancer-associated transcript-1)	↓Caspase-3, ↓MMP-2, ↓vimentin, ↓Wnt/β-catenin, ↓MEK/ERK, ↓PARP	Inhibited proliferation, spread, and infiltration of melanoma cells.	[155]
Baicalein	Human gastric carcinoma HGC-27, SGC-7901, MGC-803,and BGC-823 cell lines	0, 5, 15,25, and 50 µmol/L	24, 48, and 72 h	Untreated HGC-27, SGC-7901, MGC-803,and BGC-823 cells	miR-7/FAK/AKT signaling axis	↑miR-7, ↓FAK expression, ↓p-PI3K, ↓p-mTOR, ↓p-FAK, ↓p-AKT	Repressed gastric cancer progression, metastasis, and angiogenesis.	[156]
Baicalein	Human colon carcinoma Hct116 cell lines	0, 10, 20, and 40 μM	1–4 h	Untreated HGC-27, SGC-7901, MGC-803,and BGC-823 cells	Nrf2 (Nuclear factor erythroid 2-related factor 2) signaling axis	↓Ser40 phosphorylation, ↓NFκB	Anti-inflammatory response, induced apoptosis.	[132]
Baicalein	Human lung adenocarcinoma PC9, H1299, H1650, H358, A549, and H1975 cell lines	0, 25, 50, 75, 100, and 125 μmol/L	24, 48, and 72 h	Untreated PC9, H1299, H1650, H358, A549, and H1975 cells	AMPKα/MEK/ERK1&2/FoxO signaling axis	↑FOXO3a and RUNX3	Induced apoptosis, inhibited cell growth.	[132]

Abbreviations: METC: Mitochondrial electron transfer chain, VDAC: Voltage-dependent anion channel.

## Data Availability

Data presented within the article.
